# Perceived parental autonomy support and moral behavior in youth athletes: applying the trans-contextual model

**DOI:** 10.3389/fpsyg.2026.1780952

**Published:** 2026-03-05

**Authors:** Yeonho Choi, Youngkyun Sim, Kisun Hwang, Junsu Bae

**Affiliations:** 1 Department of Teakwondo College of Physical Education, Chosun University, Gwangju-si, Republic of Korea; 2 Department of Sports Welfare Convergence Research Institute, Woosuk University, Jeonju-si, Republic of Korea; 3 Department of Recreation and Leisure Sports, Dankook University, Yongin-si, Republic of Korea; 4 Institute of Sports Contents, Gyeongkuk National University, Andong-si, Republic of Korea

**Keywords:** antisocial behavior, intention, prosocial behavior, self-determination theory, youth sports

## Abstract

**Introduction:**

This study empirically examined the role of perceived parental autonomy support in promoting moral behavior among student-athletes through the Trans-Contextual Model (TCM).

**Methods:**

Using data from 355 Korean high school athletes across 16 sports, we investigated the effects of autonomy support on autonomous and controlled motivations in everyday and sport contexts, and their subsequent influence on moral attitudes, subjective norms, perceived behavioral control, and prosocial and antisocial behaviors.

**Results:**

Structural equation modeling revealed that parental autonomy support positively influenced both autonomous and controlled motivations in daily life, which then transferred to sport contexts. Autonomous motivation significantly predicted moral beliefs and intentions, leading to increased prosocial and decreased antisocial behaviors. Interestingly, controlled motivations also positively impacted subjective norms, suggesting complex motivational processes underlying moral conduct.

**Conclusion:**

These findings extend previous research by integrating Self-Determination Theory and the Theory of Planned Behavior, highlighting the importance of motivational quality and contextual transfer in shaping athletes’ moral actions. Practical implications for coaching, parental education, and athlete development programs are discussed, along with directions for future longitudinal and cross-cultural research.

## 1 Introduction

Sport is widely regarded as both a popular leisure activity and an effective educational tool (Ntoumanis et al., 2012). There is a consensus among researchers that participation in sports provides adolescents with opportunities for social interaction, which can contribute to the development of moral norms and values (Bruner et al., 2018; Rutten et al., 2011). Despite the common perception of sport as a context that fosters prosocial values such as sportsmanship and fair play, numerous studies have also reported negative outcomes, including increased aggression and unethical behavior (Donahue et al., 2009; Hodge and Lonsdale, 2011). In particular, within the context of elite and highly competitive sports settings, young athletes may frequently encounter improper conduct (e.g., rule-breaking, aggression). This can have adverse consequences for their development and curtail the potential of sport as a medium for moral education (Al-Yaaribi et al., 2016; Fraser-Thomas and Côté, 2009). Consequently, ascertaining the factors that influence moral behavior among youth athletes is a critical step towards promoting moral development and preventing antisocial behavior.

Within the elite sporting domain, characterized by intense competition and where future careers may be contingent on athletic performance, there is a heightened propensity for the emergence of moral issues. It is a well-documented fact that young athletes frequently find themselves confronted with moral dilemmas, necessitating a decision between achieving victory and acting in accordance with ethical principles. In such situations, athletes may either engage in morally sound behavior or resort to actions that contradict moral values (Shields and Bredemeier, 1995). In the domain of sport psychology, the concept of moral behavior in athletic contexts is conventionally theorized within a two-dimensional framework encompassing prosocial and antisocial behavior (Kavussanu and Boardley, 2009). Prosocial behavior is defined as voluntary actions intended to help or benefit others. Examples of prosocial behavior include helping an opponent who has fallen or offering congratulations after a match. Conversely, antisocial behavior encompasses deliberate actions intended to cause harm or disadvantage others, encompassing aggression and cheating. Prosocial behavior is characterized by “proactive morality,” defined as the voluntary pursuit of ethical actions. Conversely, the absence of antisocial behavior is indicative of “inhibitive morality,” which is defined as self-restraint from unethical conduct (Bandura, 1999).

In the context of moral dilemmas, some young athletes have been observed to demonstrate prosocial behavior, while others have been observed to exhibit antisocial behavior. These differences are frequently attributable to the cognitive processes that underpin athletes’ moral judgments (Jones, 2019). Since the inception of early studies in this field, cognitive frameworks – such as personal beliefs and intentions – have been considered central to understanding moral behavior (Kohlberg, 1984; Piaget, 1923; Rest, 1984). In this regard, a more profound comprehension of the intentions and beliefs that underpin prosocial or antisocial behavior in youth athletes can be facilitated by the Theory of Planned Behavior (TPB; Ajzen, 1991). The TPB posits that three belief-based components – namely, attitudes, subjective norms, and perceived behavioral control – form behavioral intentions, which in turn guide behavior. Attitude is defined as an individual’s positive or negative evaluation of a specific behavior. Subjective norms are defined as the perceived expectations of significant others (e.g., family, friends, teammates). Perceived behavioral control is defined as one’s belief in their capability and opportunity to perform the behavior. This theory has been extensively validated as a robust framework for predicting and explaining human behavior (Hagger et al., 2002).

Furthermore, the moral beliefs, intentions, and behaviors of young athletes in sport-specific contexts may be influenced by their underlying motivation. Both autonomous and controlled motivation have been shown to play key roles in shaping moral beliefs and decisions. Research indicates that athletes who exhibit high levels of autonomous motivation are more likely to perceive moral behavior as consistent with their personal values and beliefs (Deci and Ryan, 2000). These athletes tend to view prosocial behavior favorable, believing that such behavior reinforces their autonomy and internal satisfaction. Consequently, they are more likely to engage in prosocial behavior and form strong intentions to act morally (Hodge and Lonsdale, 2011). In contrast, athletes driven by controlled motivation primarily act to obtain external rewards or avoid punishment and may not necessarily align their moral actions with their personal beliefs. These individuals have been shown to be more prone to justifying antisocial behavior, resulting in weaker moral intentions (Boardley and Kavussanu, 2009). In this regard, athletes with high autonomous motivation are more likely to hold positive beliefs about moral behavior, whereas those with high controlled motivation may hold weaker or more negative moral beliefs.

While such motivational orientations may emerge within the context of sporting activities, it is crucial to recognize that the lives of young athletes are intricately interwoven with a broader range of daily experiences. Motivation cultivated in quotidian settings can transfer to sporting contexts. The Trans-Contextual Model (TCM; Hagger and Chatzisarantis, 2016) posits that motivation established in one context is likely to persist and exert influence in other contexts. For instance, autonomous or controlled motivation formed in everyday life can influence behavior in the sporting domain. TCM integrates three key theories: Self-Determination Theory (Deci and Ryan, 2000), the TPB (Ajzen, 1991), and the Hierarchical Model of Motivation (Vallerand, 2007). The fundamental premise of TCM is that autonomy-supportive figures cultivate perceived autonomy and self-determined motivation in a specific context, with the hypothesis that this motivation is likely to transcend into other contexts.

Parents represent a significant social influence on the motivation of youth athletes in their daily lives. During adolescence, the role of parents in shaping multiple aspects of development is well-documented. In particular, the degree to which parents are able to exercise autonomy in their own actions and decisions can be considered an important environmental factor affecting the choices young people make (Grolnick and Ryan, 1989). When parents respect their children’s perspectives, encourage autonomous choices, and foster intrinsic motivation, youth athletes are more likely to develop autonomous motivation in daily life (Ryan and Deci, 2000). In contrast, when parenting is characterized by an emphasis on external rewards or punishments, youth athletes may become more vulnerable to external pressures, resulting in heightened controlled motivation (Vansteenkiste et al., 2005). According to the TCM, motivation in daily life is likely to transfer into sporting contexts (Hagger et al., 2003). It can be posited that youth athletes who evince higher degrees of autonomous motivation in everyday life are more likely to sustain such motivation in sport. By contrast, those with higher degrees of controlled motivation may continue to act in accordance with external contingencies. This motivational continuity is a fundamental mechanism in comprehending moral behavior in sport.

**Figure 1:**
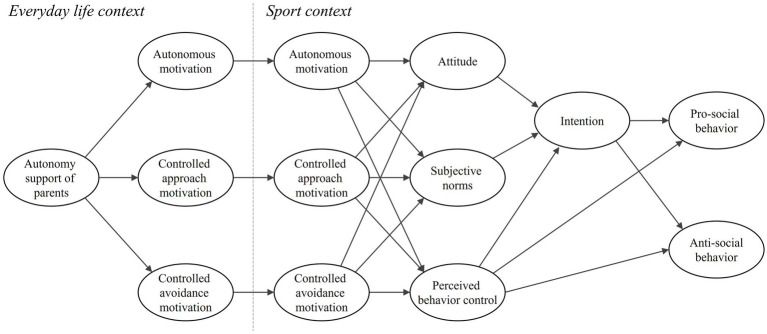
Conceptual model of this study.

In summary, the provision of parental autonomy support has the capacity to influence the motivation of youth athletes in their day-to-day lives. In accordance with the principles of TCM, it is hypothesized that such motivation is likely to transfer to the sporting context. Following the transfer process, motivation exerts a significant influence on the development of moral beliefs within the context of the sport, thereby shaping behavioral intentions. These intentions ultimately guide athletes’ decisions to engage in either prosocial or antisocial behaviors during training or competition. To date, there has been a paucity of comprehensive research that has comprehensively examined this pathway. The present study aims to address this gap by applying the TCM (Hagger and Chatzisarantis, 2016), which integrates Self-Determination Theory, the Theory of Planned Behavior, and moral behavior theory (Kavussanu and Boardley, 2009), to better understand how youth athletes’ moral actions are formed. It is anticipated that this approach will yield valuable insights and make a significant contribution to the academic literature. The purpose of this study is to investigate how perceived parental autonomy support in daily life influences youth athletes’ moral behavior in sport through the processes outlined in the TCM. The proposed model is illustrated in Figure 1.

## 2 Methods

### 2.1 Participants

The participants comprised 355 student-athletes from South Korea, including 202 males (56.9%) and 153 females (43.1%). All participants were affiliated with high school sports teams and were officially registered as student-athletes under the Korea Olympic Committee (KOC). The participants of the study ranged in age from 16 to 18 years, corresponding to high school age in Korea, with a mean age of 16.99 years (*SD* = 0.83). The mean duration of experience competing as student-athletes was 4.28 years (*SD* = 1.83; range = 1–8). The participants represented 16 different sports. The most prevalent sport was taekwondo (*n* = 80, 22.5%), followed by soccer (*n* = 50, 14.1%), shooting (*n* = 36, 10.1%), swimming (*n* = 27, 7.6%), boxing and wrestling (*n* = 23 each, 6.5%), judo and track and field (*n* = 19 each, 5.4%), and modern pentathlon (*n* = 17, 4.8%), among other sports.

### 2.2 Measures

#### 2.2.1 Parental autonomy support

The measurement of parental autonomy support as perceived by the athletes was conducted using the Korean short version of the scale originally developed by Soenens et al. (2007) and validated by Moon and Lee (2022). The instrument under scrutiny comprises two subscales, with a total of eight items each. The first subscale pertains to the promotion of independence, with items such as “My parents encourage me to think independently”. The second subscale relates to the promotion of volitional functioning, with items such as “My parents allow me to choose the direction of my own life”. Each subscale consists of four items. Responses were evaluated using a 5-point Likert scale, ranging from 1 (not at all true) to 5 (very true). In the present study, the internal consistency was satisfactory, with Cronbach’s alpha coefficients of 0.707 for the promotion of independence subscale and 0.837 for the promotion of volitional functioning subscale.

#### 2.2.2 Autonomous and controlled motivation

The assessment of autonomous and controlled motivation was conducted utilizing the Korean version of the Prosocial Self-Regulation Questionnaire (SRQ-P; Ryan and Connell, 1989), which was adapted by Lee (2024) based on Self-Determination Theory. The present instrument is designed to evaluate the underlying motivations for four distinct prosocial behaviors: caring, kindness, yielding, and rule-following. The scale comprises 8 items for autonomous motivation, 4 items for controlled approach motivation, and 8 items for controlled avoidance motivation. The contextualization of the stem items was achieved through the utilization of expert consultation, thereby ensuring their relevance within both quotidian life and sporting contexts. For instance, in the context of daily life, the stem proffered was, “The reason I act kindly towards others in daily life is because…”, while in the context of sporting activities, the stem posed was, “The reason I act kindly towards teammates or opponents during practice or competition is because…”. The items were evaluated using a 5-point Likert scale, ranging from 1 (not at all true) to 5 (very true). In the context of daily life, the reliability coefficients were 0.895 for autonomous motivation, 0.900 for controlled approach motivation, and 0.914 for controlled avoidance motivation. In the context of sporting activities, the reliability coefficients were determined to be 0.924 for autonomous motivation, 0.928 for controlled approach motivation, and 0.913 for controlled avoidance motivation.

#### 2.2.3 Beliefs and intentions

The TPB was utilized as a framework to measure beliefs and intentions (Ajzen, 2002). Context-specific items were developed in accordance with TPB guidelines to facilitate the measurement process. The central stem for assessing moral intention, subjective norms, and perceived behavioral control was formulated as follows: “When training or competing, acting kindly towards others is something I…”. Participants were instructed to respond to three intention items (e.g., “…plan to do”), four subjective norm items (e.g., “…is expected of me by people around me”), and five perceived behavioral control items (e.g., “…can do if I choose to”). The items were evaluated using a 5-point Likert scale, ranging from 1 (not at all true) to 5 (very true). The participants’ attitudes towards moral behavior in sport were measured using a bipolar semantic differential format with six items based on the stem, “When I act kindly towards others during training or competition, it is…”. The scale ranges from 1, representing “useless,” to 5, representing “useful”. The Cronbach’s alpha coefficients for each construct were as follows: attitude 0.918, subjective norm 0.890, perceived behavioral control 0.882, and intention 0.863.

#### 2.2.4 Moral behavior in sport

The moral behavior of athletes was measured using the Korean version of the scale developed by Kavussanu and Boardley (2009), which was adapted by Jang et al. (2016). The present instrument is designed to assess behaviors that are both prosocial and antisocial, with regard to both teammates and opponents. The scale comprises four subscales: prosocial behavior towards teammates (4 items), antisocial behavior towards teammates (5 items), prosocial behavior towards opponents (3 items), and antisocial behavior towards opponents (8 items), with a total of 20 items. The rating of each item was conducted using a 5-point Likert scale, ranging from 1 (not at all true) to 5 (very true). The following coefficients were obtained for the subscales: The mean values for prosocial behavior towards teammates were 0.837, for antisocial behavior towards teammates 0.882, for prosocial behavior towards opponents 0.739, and for antisocial behavior towards opponents 0.951.

### 2.3 Procedure

A non-probability sampling method was employed to recruit student-athletes from high school athletic teams in South Korea. Following the preliminary coordination with the school’s administrative body, the research team proceeded to liaise with coaches or team supervisors, requesting their collaboration in the collection of data. For teams that agreed to participate, official letters and parental informed consent forms were distributed to the athletes’ guardians (e.g., parents) in order to obtain approval. After obtaining informed consent from the parents or guardians of all minor participants, researchers visited the schools and distributed informed consent forms and questionnaires to student-athletes who voluntarily agreed to participate. In accordance with the standards for ethical research, participants were informed about the anonymity of the survey, their rights to access or withdraw their data, the intended use of the data, and the voluntary nature of their participation. The completion of the questionnaires was conducted through self-report, with the data collected immediately upon completion. To encourage sincere participation, a small token of appreciation was provided to the athletes.

### 2.4 Data analysis

The collected data were analyzed using IBM SPSS version 23 and AMOS version 23. Initially, the distribution of the data was examined using descriptive statistics, which included the mean, standard deviation, skewness, and kurtosis. The thresholds for assessing normality were set at an absolute value of 3 for skewness and 8 for kurtosis (Kline, 2023). Subsequently, Pearson’s product–moment correlation analysis was conducted to explore the bivariate relationships among the study variables. Following conventional guidelines, correlation coefficients below 0.90 indicate that multicollinearity is unlikely to pose a serious problem (Tabachnick and Fidell, 2019). In order to test the hypothesized relationships, a structural equation modeling (SEM) approach was employed. The model fit was assessed using the following indices: the chi-square divided by degrees of freedom (*χ*^2^/*df*), the Comparative Fit Index (CFI), the Tucker–Lewis Index (TLI), the Root Mean Square Error of Approximation (RMSEA), and the Standardized Root Mean Square Residual (SRMR). According to Kline (2023), a model is considered to demonstrate a good fit when the chi-squared statistic divided by the degrees of freedom (*χ*^2^/*df*) is below 3, the comparative fit index (CFI) and the Tucker–Lewis index (TLI) are above 0.90, and the root mean square error of approximation (RMSEA) and the standardized root mean square residual (SRMR) are below 0.08.

## 3 Results

### 3.1 Descriptive statistics and correlations

Table 1 presents the descriptive statistics and bivariate correlations for all measured variables. The mean scores ranged from 1.329 to 4.242, with standard deviations ranging from 0.460 to 0.998. Tests for normality indicated that none of the measured variables exceeded the recommended thresholds of skewness (±3) and kurtosis (±8), suggesting that the data met the assumptions of normal distribution (Kline, 2023). With regard to the correlations among the variables, several subscales were found to be significantly correlated. The lowest bivariate correlation was observed between moral intention and antisocial behavior towards teammates (*r* = −0.405, *p* < 0.01), while the highest correlation was found between controlled approach motivation in daily life (L_CAP) and in sport contexts (S_CAP), with *r* = 0.838 (*p* < 0.01). It is important to note that controlled avoidance motivation in sport contexts (S_CAV) demonstrated no significant correlations with any of the four dimensions of moral behavior in sport. Regarding multicollinearity, although several factors exhibited relatively high correlation coefficients, none exceeded 0.90 (Tabachnick and Fidell, 2019); therefore, multicollinearity was not considered to be a serious concern.

**Table 1:** Descriptive statistics and bi-correlations coefficients of all sub-factors.

Factor	1	2	3	4	5	6	7	8	9	10	11	12	13	14	15	16
1. PI	1															
2. PVF	0.439^**^	1														
3. L_AM	0.241^**^	0.328^**^	1													
4. L_CAP	0.115^*^	0.141^**^	0.495^**^	1												
5. L_CAV	0.001	−0.095	0.104	0.400^**^	1											
6. S_AM	0.284^**^	0.359^**^	0.709^**^	0.438^**^	0.053	1										
7. S_CAP	0.078	0.110^*^	0.434^**^	0.838^**^	0.340^**^	0.489^**^	1									
8. S_CAV	−0.034	−0.112^*^	0.061	0.323^**^	0.833^**^	0.081	0.361^**^	1								
9. Attitude	0.114^*^	0.183^**^	0.418^**^	0.288^**^	0.070	0.451^**^	0.328^**^	0.090	1							
10. Subjective norms	0.177^**^	0.163^**^	0.442^**^	0.392^**^	0.203^**^	0.478^**^	0.450^**^	0.184^**^	0.467^**^	1						
11. PBC	0.302^**^	0.325^**^	0.531^**^	0.247^**^	−0.006	0.604^**^	0.277^**^	0.016	0.423^**^	0.449^**^	1					
12. Intention	0.125^*^	0.212^**^	0.594^**^	0.399^**^	0.045	0.621^**^	0.427^**^	0.059	0.529^**^	0.612^**^	0.561^**^	1				
13. TP	0.265^**^	0.369^**^	0.532^**^	0.292^**^	−0.022	0.544^**^	0.310^**^	0.003	0.283^**^	0.337^**^	0.469^**^	0.459^**^	1			
14. OP	0.250^**^	0.217^**^	0.377^**^	0.239^**^	0.081	0.396^**^	0.233^**^	0.056	0.419^**^	0.403^**^	0.368^**^	0.487^**^	0.364^**^	1		
15. TA	−0.043	−0.121^*^	−0.338^**^	−0.163^**^	0.103	−0.328^**^	−0.223^**^	0.087	−0.244^**^	−0.248^**^	−0.357^**^	−0.405^**^	−0.267^**^	−0.203^**^	1	
16. OA	−0.097	−0.151^**^	−0.192^**^	−0.088	0.106^*^	−0.305^**^	−0.165^**^	0.052	−0.260^**^	−0.165^**^	−0.318^**^	−0.307^**^	−0.158^**^	−0.274^**^	0.546^**^	1
Mean	3.581	4.169	4.068	3.534	2.589	4.132	3.371	2.494	4.154	3.549	4.242	3.975	4.012	3.388	1.631	1.329
Standard deviation	0.689	0.653	0.555	0.889	0.902	0.577	0.998	0.910	0.689	0.902	0.639	0.779	0.627	0.796	0.611	0.460
Skewness	−0.090	−0.703	−0.361	−0.291	0.362	−0.206	−0.072	0.581	−0.461	−0.296	−0.583	−0.545	−0.119	−0.118	0.507	1.011
Kurtosis	0.098	0.474	0.627	−0.233	−0.086	−0.190	−0.721	−0.293	−0.519	0.042	0.137	0.722	−0.433	0.578	−0.714	−0.428

^*^p < 0.05, ^**^p < 0.01.

PI, Promotion of independence; PVF, Promotion of volitional functioning; L_AM, Autonomous motivation in life; L-CAV, Controlled avoidance motivation in life; L_CAP, Controlled approach motivation in life; S_AM, Autonomous motivation in sport; S-CAV, Controlled avoidance motivation in sport; S_CAP, Controlled approach motivation in sport; PBC, Perceived behavior control; TP, Team pro-social behavior; OP, Opponent pro-social behavior; TA, Team anti-social behavior; OA, Opponent anti-social behavior.

### 3.2 Structural equation model

Table 2 presents the results of the structural equation model (SEM) analysis. The model can be interpreted in three key domains: (a) the daily life context, (b) the contextual transfer from daily life to sport, and (c) the sport-specific context.

**Table 2:** Estimates and standardized estimates of all paths.

Paths	*B*	*β*	S.E.	*t*	*p*
Daily life context					
AS → L_AM	1.686	0.906	0.297	5.674	^***^
AS → L_CAV	0.493	0.159	0.202	2.440	0.015
AS → L_CAP	2.020	0.604	0.320	6.309	^***^
Contextual transfer					
L_AM → S_AM	0.893	0.870	0.065	13.746	^***^
L_CAP → S_CAP	0.973	0.916	0.048	20.390	^***^
L_CAV → S_CAV	0.966	0.926	0.050	19.476	^***^
Sports context					
S_AM → Attitude	0.669	0.520	0.082	8.163	^***^
S_AM → Subjective norms	0.835	0.490	0.103	8.078	^***^
S_AM → PBC	0.868	0.771	0.075	11.506	^***^
S_CAP → Attitude	0.059	0.086	0.039	1.536	0.124
S_CAP → Subjective norms	0.177	0.193	0.050	3.542	^***^
S_CAP → PBC	−0.039	−0.065	0.030	−1.299	0.194
S_CAV → Attitude	0.015	0.020	0.038	0.396	0.692
S_CAV → Subjective norms	0.110	0.110	0.049	2.271	0.023
S_CAV → PBC	−0.032	−0.048	0.029	−1.090	0.276
Attitude → Intention	0.275	0.257	0.053	5.165	^***^
Subjective norms → Intention	0.216	0.267	0.041	5.288	^***^
PBC → Intention	0.527	0.430	0.070	7.503	^***^
PBC → Pro-social behavior	0.311	0.480	0.057	5.442	^***^
PBC → Anti-social behavior	−0.245	−0.297	0.067	−3.685	^***^
Intention → Pro-social behavior	0.400	0.814	0.047	8.457	^***^
Intention → Anti-social behavior	−0.350	−0.514	0.046	−7.615	^***^

^***^p < 0.001.

AS, Autonomy support; L_AM, Autonomous motivation in daily life; L_CAV, Controlled avoidance motivation in daily life; L_CAP, Controlled approach motivation in daily life; S_AM, Autonomous motivation in sport; S_CAV, Controlled avoidance motivation in sport; S_CAP, Controlled approach motivation in sport; PBC, Perceived behavior control.

#### 3.2.1 Daily life context

In the domain of daily life, parental autonomy support (AS) significantly predicted all three types of athlete motivation: autonomous motivation (L_AM), controlled avoidance motivation (L_CAV), and controlled approach motivation (L_CAP). Specifically, parental autonomy support had a strong positive effect on autonomous motivation (*β* = 0.906, *t* = 5.674, *p* < 0.001), a modest effect on controlled avoidance motivation (*β* = 0.159, *t* = 2.440, *p* < 0.05), and a substantial effect on controlled approach motivation (*β* = 0.604, *t* = 6.309, *p* < 0.001).

#### 3.2.2 Contextual transfer: from daily life to sport

All three types of motivation in daily life significantly influenced their corresponding types of motivation in the sport context. Autonomous motivation in daily life positively predicted autonomous motivation in sport (S_AM) (*β* = 0.870, *t* = 13.746, *p* < 0.001). Controlled approach motivation in daily life strongly predicted controlled approach motivation in sport (S_CAP) (*β* = 0.916, *t* = 20.390, *p* < 0.001), and controlled avoidance motivation in daily life significantly predicted controlled avoidance motivation in sport (S_CAV) (*β* = 0.926, *t* = 19.476, *p* < 0.001).

#### 3.2.3 Sport context

**Figure 2:**
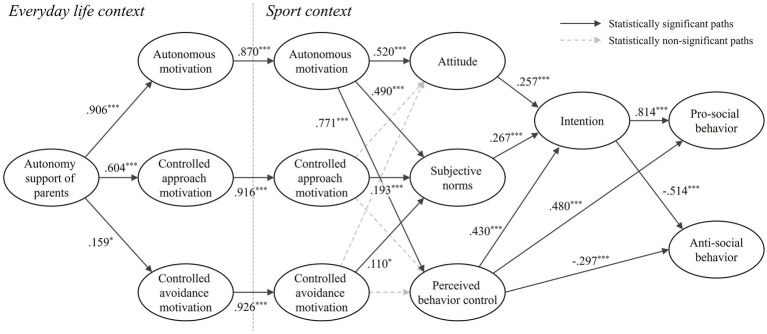
Tested research model.

Within the sport context, the three types of motivation had differential effects on the components of the TPB. Autonomous motivation in sport significantly influenced attitude (*β* = 0.520, *t* = 8.163, *p* < 0.001), subjective norm (*β* = 0.490, *t* = 8.078, *p* < 0.001), and perceived behavioral control (*β* = 0.771, *t* = 11.506, *p* < 0.001). Controlled approach motivation in sport had a significant effect only on subjective norm (*β* = 0.193, *t* = 3.542, *p* < 0.001), and controlled avoidance motivation in sport also affected only subjective norm (*β* = 0.110, *t* = 2.271, *p* < 0.05). Regarding intention, all three TPB components—attitude (*β* = 0.257, *t* = 5.165, *p* < 0.001), subjective norm (*β* = 0.267, *t* = 5.288, *p* < 0.001), and perceived behavioral control (*β* = 0.430, *t* = 7.503, *p* < 0.001)—significantly and positively predicted athletes’ intentions to engage in moral behavior. Notably, perceived behavioral control also had significant effects on behavior: it positively predicted prosocial behavior (*β* = 0.480, *t* = 5.442, *p* < 0.001) and negatively predicted antisocial behavior (*β* = −0.297, *t* = −3.685, *p* < 0.001). Finally, moral intention significantly predicted moral behavior: it had a strong positive effect on prosocial behavior (*β* = 0.814, *t* = 8.457, *p* < 0.001) and a significant negative effect on antisocial behavior (*β* = −0.350, *t* = −7.615, *p* < 0.001). Figure 2 presents the final structural model with analyzed data. In the figure, statistically significant paths are represented by solid lines, whereas non-significant paths are indicated by dashed lines to provide a clearer distinction of the empirical results.

## 4 Discussion

The present study empirically examined the pathways from parental autonomy support perceived by student-athletes to their moral behavior in the sporting context, utilizing the TCM. Of particular note was the manner in which it addressed autonomous motivation, controlled approach motivation, and controlled avoidance motivation in the context of prosocial behavior. The findings indicated that parental autonomy support significantly influenced all three motivational types in the daily life context. In addition, it was demonstrated that these three forms of motivation were directly transferable to the sporting context. Autonomous motivation was found to exert a significant positive influence on the TPB components, namely attitude, subjective norm, and perceived behavioral control. This, in turn, resulted in increased prosocial behavior and decreased antisocial behavior. The findings of this study imply that moral behavior is not confined to a single context, but rather is intricately linked to the broader motivational system that pervades an athlete’s life. In other words, moral actions can be considered as the result of long-term motivational processes, such as the motivational climate in the family environment, rather than short-term situational judgments. This structural pathway contributes to a more nuanced explanation of the mechanisms underlying moral behavior and provides a novel theoretical foundation for research on morality in sport psychology.

A significant theoretical contribution of this study is the use of structural equation modeling to statistically confirm the complex sequence of parental autonomy support → autonomous motivation → TPB components → moral behavior. Contrary to the prevailing focus of earlier research on moral behavior, which was often oriented towards situational factors or discrete cognitive components, this study conceptualizes motivational continuity and contextual transfer as pivotal theoretical constructs. This approach extends the two-dimensional prosocial–antisocial behavior framework proposed by Kavussanu and Boardley (2009), demonstrating how motivational backgrounds significantly influence the qualitative nature of behavior. Furthermore, the demonstration of the formation of belief structures in TPB according to motivational quality serves to both reconfirm and extend Ajzen (1991)’s theory within the context of sporting moral behavior. This integrative, multi-theoretical approach facilitates a comprehensive understanding of psychological variables and offers a methodological model capable of explaining causal relationships among diverse factors with greater precision.

A more thorough examination of the results reveals that parental autonomy support has a positive impact on both autonomous motivation and controlled approach and avoidance motivations. From the perspective of Self-Determination Theory, this finding is somewhat unexpected, as autonomy support has generally been understood to negatively correlate with controlled motivation (Deci and Ryan, 2000; Vansteenkiste et al., 2005). This outcome prompts several interpretive considerations. In reality, parental autonomy support may not always manifest as a consistent or purely intrinsic motivational facilitator. Concomitant encouragement of autonomy by parents, alongside the maintenance of elevated achievement expectations or the provision of outcome-focused feedback, has the potential to engender controlled motivation in the athlete (Mageau et al., 2009). Furthermore, adolescent student-athletes frequently encounter intricate psychological interpretations within the tension between parental expectations and the competitive sports environment. For instance, even if parents provide an autonomy-supportive environment, athletes may perceive this as pressure to perform well independently or as a responsibility to avoid disappointing parental trust. In the Korean cultural context, this phenomenon can be further explained by the concept of ‘filial piety (Hyo)’. Even when parents provide an autonomy-supportive environment, Korean youth athletes may internalize their parents’ support as a form of ‘benevolent pressure,’ feeling a strong sense of obligation to succeed as a way of repaying parental devotion. This may result in the internalization of controlled motivation (especially introjected regulation) (Ryan and Connell, 1989). This finding aligns with the broad perspective of Self-Determination Theory, which posits that autonomy and control exist on a continuous scale and that motivational internalization is multifaceted (Sheldon et al., 2004). The finding may also be partially accounted for by cultural differences in autonomy (Miller et al., 2002) and interpretative variations in measurement items. The positive influence of parental autonomy support on both autonomous and controlled motivations, as evidenced in this study, can be interpreted as indicative of the intricacy and heterogeneity of motivational internalization processes.

A further salient finding is that both controlled approach and avoidance motivations in the sporting context positively influenced subjective norm. This finding is consistent with previous research that has demonstrated a positive correlation between autonomous motivation and subjective norm. However, it also suggests the possibility that controlled motivation may be positively associated with perceptions of others’ expectations and social norms (Chan et al., 2015; Hagger et al., 2002). Controlled motivation is typically understood to be a driving force behind behavior that is influenced by external pressures as opposed to being internally motivated by individuals’ own beliefs or values. This concept posits that individuals tend to be more influenced by external standards or expectations. In this regard, subjective norm – reflecting sensitivity to others’ expectations – may theoretically exhibit a positive relationship with controlled motivation. For instance, student-athletes who exhibit controlled approach motivation may act with the intention of gaining praise, recognition, or rewards, thereby naturally heightening their sensitivity to the evaluations or social expectations of others. This psychological structure corresponds to the tendency to respond strongly to subjective norms (“others think I should behave this way”), even if these are not fully internalized values. In such cases, subjective norms are likely to function as external pressures rather than being internalized (Ryan and Connell, 1989).

In a similar manner, the concept of controlled avoidance motivation, which is driven by an individual’s desire to evade punishment or negative evaluation, has the capacity to render athletes acutely sensitive to the perceptions and social standards of others. This phenomenon is especially pertinent in team sports and organized training systems, where motivations such as adhering to rules to evade criticism or exercising caution to avoid disturbing teammates are prevalent adaptive strategies. These avoidance motivations may represent strategic behavioral adaptations aimed at avoiding negative feedback rather than internalized norms, thus explaining their association with subjective norm sensitivity. This finding suggests that subjective norm does not necessarily serve as an indicator of autonomous internalization; rather, it may be associated with controlled motivation. Consequently, perceptions of social norms may vary qualitatively depending on motivation type, with compliance driven either autonomously or by external pressures. This study suggests that a more nuanced interpretation of the subjective norm construct within TPB is required, and it is recommended that future research explore the motivational reasons behind norm adherence. However, the positive relationship between controlled motivation and subjective norms raises concerns regarding the sustainability of moral behavior. If athletes engage in prosocial behavior primarily to satisfy social expectations or avoid negative evaluations (i.e., internalized conformity), these actions may not persist when external monitoring or social pressures are absent. This highlights that while subjective norms can lead to immediate moral compliance, long-term moral development must be rooted in autonomous internalization rather than externalized social pressure.

Finally, given that sporting environments inherently emphasize discipline, directives and evaluation, it is important to recognize that such contexts may strengthen controlled motivation. It is important to note that athletes may engage in prosocial behavior not out of autonomous choice, but rather as a means to meet the expectations of their team, their coach, or to avoid the consequences of peer pressure. While this approach can yield favorable short-term outcomes, behavior that is contingent on norm compliance without autonomous internalization is susceptible to alteration in the event of shifting circumstances. This has the potential to erode the sustainability of moral behavior over time.

Parental autonomy support is a crucial socialization factor in itself, but this study adds educational implications by demonstrating its linkage to moral behavior outcomes. Grolnick and Ryan (1989)’s seminal study observed the close associations between autonomy support and self-regulated learning, as well as emotional stability. The present findings extend this understanding to the specific behavioral domain of sporting moral conduct, thereby reaffirming the critical role of parents during adolescence. These insights can inform practical interventions in the following areas: athlete education, parent counselling and school sport policy. This is particularly salient for student-athletes, whose moral judgment is critical to their development. Parental style should therefore be considered a strategic factor that extends beyond mere support.

## 5 Field application

The findings of this study provide several meaningful implications for practical settings aimed at promoting moral behavior among student-athletes. Firstly, it is evident that parental autonomy support exerts a substantial influence on the overarching motivational framework of student-athletes. Consequently, the scope of moral education and sport ethics training should not be confined solely to the athletes themselves. In order to effectively enhance positive moral behavior, there is a necessity for programs to be designed with the aim of educating and supporting key caregivers, including parents, in adopting autonomy-supportive parenting styles. Specifically, parents can adopt an autonomy-supportive communication style by using non-pressuring language and acknowledging their child’s feelings. Instead of outcome-oriented comments like ‘You should be more polite to others to get a good reputation,’ parents could ask, ‘How did you feel when you helped your teammate today?’ or ‘What do you think is the most respectful way to treat your opponent in that situation?’. Providing such rationales and choices helps athletes internalize moral values as their own, rather than following them as external rules.

Furthermore, the role of coaches and instructors within the sporting environment is of particular significance in promoting ethical conduct among athletes. As corroborated by the present study, athletes’ autonomous motivation exerts a positive influence on their attitudes, normative beliefs, and perceived behavioral control, consequently resulting in heightened prosocial behavior. Consequently, coaches must cultivate autonomy-supportive coaching styles. It is recommended that sport associations and the Korea Sports Council develop and implement ‘morality-centered coaching education programs,’ incorporating autonomy-focused motivational theory and practical case studies into mandatory annual training. The analysis and discussion of the influence of verbal expressions, reinforcement methods and feedback styles in coaching situations on athletes’ motivational types, using real video materials, could serve as effective instructional strategies.

Finally, the study posits that morality and motivation should be incorporated into the selection and career guidance processes of athletes. This approach serves to diversify the definition of a ‘good athlete’ beyond mere performance and contributes to the fostering of a healthy sporting culture. For instance, in the context of school sports club competitions or elite sports leagues, the establishment of evaluation systems is imperative to assess and visualize moral components such as teamwork, consideration, and cooperation, in addition to athletic performance. The implementation of such systems is poised to facilitate a tangible appreciation among athletes of the value of moral behavior, thereby reinforcing morality as an internalized belief rather than an external norm.

## 6 Future direction

In order to further advance the existing literature on this subject, future research must address the limitations of the present study. Firstly, an exploratory approach to the heterogeneous coexistence of motivation types is necessary. The present study found that parental autonomy support positively influenced both autonomous motivation and controlled motivation. This suggests that contrasting types of motivation can coexist within the same context. It is recommended that future studies employ in-depth qualitative research to explain this heterogeneous coexistence of motivational structures. For instance, conducting in-depth interviews with student-athletes could assist in elucidating the psychological mechanisms through which autonomy and control are experienced concurrently. Furthermore, the utilization of a mixed methods design, which integrates the structure of quantitative data with the meaning of qualitative data, is recommended.

Secondly, longitudinal designs are required to temporally track the transfer of motivation. The cross-sectional design of the study limits the extent to which causal sequences in motivational transfer can be elucidated. It is recommended that future research adopts longitudinal approaches in order to trace how motivation formed in everyday contexts changes in sporting contexts over time and how this changes the influence on moral behavior. In particular, a longitudinal approach would allow for the verification of the stability and directionality of the motivation ‘transfer’ process, which is central to the TCM framework, but cannot be fully captured through cross-sectional data. Furthermore, the reliance on self-report measures for moral behavior may have introduced social desirability bias, where athletes might over-report prosocial actions and under-report antisocial ones to present themselves in a more favorable light. Future studies should incorporate multi-source assessments, such as peer-ratings or coach-evaluations, to ensure a more objective measurement of moral conduct in sports settings. In addition, future studies could employ direct observation methods or behavioral coding during actual training or competition to minimize the reliance on self-perception and capture real-time moral reactions.

Thirdly, it is necessary to clarify the relationship between types of motivation and the internalization of norms. The finding that controlled motivation also positively influences subjective norms suggests that reasons for norm compliance may vary by motivation type. It is recommended that future research extends Ajzen’s TPB by conducting a more detailed examination of the interactions between the degree of norm internalization and motivation types. In order to surmount the limitation of measuring subjective norms as a unidimensional construct in TPB, multidimensional normative components such as moral norms, legal norms and social expectations should be incorporated.

Finally, comparative research across a range of sports and cultural contexts should be expanded. As this study focused on Korean high school athletes, the results may be considered as applicable to that particular cultural and sporting context. Future studies should aim to compare the structure of autonomy support, motivation types, and moral behavior across a range of sporting activities (for example, individual vs. team sports) or cultural regions (for example, Western vs. East Asian cultures). Collectivist cultures in East Asia, for instance, might demonstrate stronger influences of subjective norms, whereas individualistic Western cultures might place relatively more emphasis on autonomous motivation (Hagger et al., 2007). The utilization of comparative research in this context confers a distinct advantage, namely the capacity to evaluate both the universality and the contextual fit of the theoretical framework. Furthermore, verifying the proposed model in individualistic cultures, such as those in Western Europe or North America, would be essential to validate the universality of the current findings and to understand how cultural differences shape the role of subjective norms.

## 7 Conclusion

This study demonstrates that perceived parental autonomy support functions as a critical socialization mechanism shaping youth athletes’ moral behavior. By applying the Trans-Contextual Model, we show that autonomy-supportive parenting fosters both autonomous and controlled motivations in daily life that transfer to sport contexts and act on athletes’ attitudes, subjective norms and perceived behavioral control. In particular, autonomous motivation emerges as the strongest predictor of prosocial intentions and behaviors, reinforcing moral beliefs and reducing antisocial conduct, whereas controlled motivations contribute indirectly by strengthening subjective norms and highlighting the nuanced role of external expectations in moral decision-making.

Moreover, our findings indicate that perceptions of behavioral control and moral intentions mediate the translation of beliefs into actual prosocial or antisocial acts. By integrating Self-Determination Theory, the Theory of Planned Behavior and moral behavior frameworks into a single structural model, this research offers a unified lens for understanding how motivational quality and contextual transfer jointly shape youth athletes’ moral actions. Practically, the results underscore the need for autonomy-supportive environments across home and sport settings, suggesting that coaching strategies, parental education and sport policies should prioritise autonomy, competence and relatedness to foster sustainable moral development.

## Data Availability

The raw data supporting the conclusions of this article will be made available by the authors, without undue reservation.
